# Magnetic Phase Diagram of the Mn*_x_*Fe_2−*x*_P_1−*y*_Si*_y_* System

**DOI:** 10.3390/e24010002

**Published:** 2021-12-21

**Authors:** Xinmin You, Michael Maschek, Niels Harmen H. van Dijk, Ekkes Brück

**Affiliations:** Fundamental Aspects of Materials and Energy (FAME), Faculty of Applied Sciences, Delft University of Technology, Mekelweg 15, 2629 JB Delft, The Netherlands; x.you-1@tudelft.nl (X.Y.); M.Maschek@tudelft.nl (M.M.); n.h.vandijk@tudelft.nl (N.H.H.v.D.)

**Keywords:** magnetocaloric materials, Mn-Fe-P-Si, phase diagram, transition temperature, thermal hysteresis, magnetic entropy

## Abstract

The phase diagram of the magnetocaloric Mn*_x_*Fe_2−*x*_P_1−*y*_Si*_y_* quaternary compounds was established by characterising the structure, thermal and magnetic properties in a wide range of compositions (for a Mn fraction of 0.3 ≤ *x* < 2.0 and a Si fraction of 0.33 ≤ *y* ≤ 0.60). The highest ferromagnetic transition temperature (Mn_0.3_Fe_1.7_P_0.6_Si_0.4_, *T_C_* = 470 K) is found for low Mn and high Si contents, while the lowest is found for low Fe and Si contents (Mn_1.7_Fe_0.3_P_0.6_Si_0.4_, *T_C_* = 65 K) in the Mn*_x_*Fe_2−*x*_P_1−*y*_Si*_y_* phase diagram. The largest hysteresis (91 K) was observed for a metal ratio close to Fe:Mn = 1:1 (corresponding to *x* = 0.9, *y* = 0.33). Both Mn-rich with high Si and Fe-rich samples with low Si concentration were found to show low hysteresis (≤2 K). These compositions with a low hysteresis form promising candidate materials for thermomagnetic applications.

## 1. Introduction

The application or removal of an external magnetic field on a magnetic solid under adiabatic conditions triggers a temperature change that is known as the magnetocaloric effect (MCE) [1]. In an isothermal process, this MCE is associated with magnetic entropy change. There are two main applications of the MCE. The first application is magnetic refrigeration, where heat is removed in a magnetic field cycle [2]. Compared to conventional gas compression refrigeration, magnetic refrigeration shows advantages, for instance, no harmful gases are released, and a high cooling efficiency can be achieved [3]. The second application is the thermomagnetic motor/generator, where waste heat is converted into mechanical/electric energy [4,5].

The key to convert current thermomagnetic motors/generators into a cost-effective application is to optimise the magnetocaloric materials. Pecharsky and Gschneidner at Ames Lab discovered a giant magnetocaloric effect in Gd_5_Si_2_Ge_2_ alloys near ambient temperature [6,7,8]. Since then, several intermetallic material systems with a giant MCE have been discovered and developed, including La(Fe,Si)_13_-based alloys [9,10,11], MnFeP(As,Ge,Si) alloys [12,13,14,15,16] and Ni–Mn-based Heusler alloys [17,18,19,20]. Among these magnetocaloric families, the (Mn,Fe)_2_(P,Si)-type compounds have been considered as one of the most promising candidates for near-room temperature magnetic refrigeration and magnetic energy conversion applications due to their combination of a tuneable working temperature, low hysteresis, corrosion resistance, compositional stability and low material costs.

In the last decade, (Mn,Fe)_2_(P,Si) compounds with varying Si-content have been thoroughly investigated for their giant MCE based on the earth-abundant and non-toxic elements [21,22,23,24,25,26,27,28]. The Mn-Fe-P-Si composition of these materials may have a distinct effect on the structural and magnetic properties. In addition, the introduction of small atoms like boron, carbon and nitrogen can also be used to tailor the magnetoelastic transition for the (Mn,Fe)_2_(P, Si) compounds while preserving a giant MCE [29,30,31,32].

Dung and co-workers [33] have constructed partial phase diagrams of the (Mn,Fe)_2_(P,Si) compounds and illustrated the composition dependence of the Curie temperature *T*_C_ (K) and the thermal hysteresis Δ*T*_hys_ for the Mn_x_Fe_2−x_P_1−y_Si_y_ compounds (*x* = 1.10–1.30, *y* = 0.50–0.58). It has been found that an increase in the Mn:Fe atomic ratio causes a reduction in both *T*_C_ (K) and in thermal hysteresis Δ*T*_hys_, while an increase in the P:Si atomic ratio leads to a decrease in *T*_C_ and an increase in thermal hysteresis. This study focused on a limited region of the Fe-Mn-P-Si phase diagram. A revised FeMnP_1−*y*_Si*_y_* phase diagram was later presented by Höglin [34], which consists of five main zones featuring two single phase regions for the orthorhombic Co_2_P-type (*y* < 0.15) structure and the hexagonal Fe_2_P-type (0.24 ≤ *x* ≤ 0.50) structure.

In the present study, an extended phase diagram of the Mn*_x_*Fe_2−*x*_P_1−*y*_Si*_y_* system is presented, with focus on the magnetocaloric active Fe_2_P-phase for a wide range of compositions (for Mn fraction of 0.3 < *x* < 2.0 and a Si fraction of 0.33 ≤ *y* ≤ 0.6). This extended phase diagram enables the search of suitable compositions for magnetic refrigeration and energy conversion applications. Furthermore, the presence of impurity phase(s) and the relationship between the heat of transformation of the magnetic transition and thermal hysteresis are discussed.

## 2. Materials and Methods

Polycrystalline Mn*_x_*Fe_2−*x*_P_1−*y*_Si*_y_* samples have been prepared by ball milling the starting materials Fe (99.9%), Mn (99.9%), red-P (99.7%) and Si (99.9%) powder. After 10 h of ball milling, the samples were pressed into tablets [14]. The tablets were sealed under Ar atmosphere in quartz ampoules, sintered at 1373 K for 25 h and then quenched into water.

Powder diffraction patterns were collected in a PANalytical X-pert Pro diffractometer with Cu *K*_α_ radiation. The lattice parameters and the impurity phases were obtained by a full Rietveld analysis of the X-ray diffraction data using the FULLPROF package [35]. The heat capacity and heat of transformation were measured in a differential scanning calorimeter (DSC). The measurements were carried out using a TA Q2000 DSC, which uses liquid nitrogen to cool the system. The sweeping rate was 10 K/min.

The magnetic properties were measured in a Superconducting Quantum Interface Device (SQUID) magnetometer (Quantum Design MPMS XL) using the RSO mode in the temperature range of 5–370 K with a sweep rate of 2 K/min and vibrating sample magnetometer (VSM) (Quantum Design VersaLab) in the temperature range of 300–600 K with a sweep rate of 10 K/min. From the magnetic measurements, the ferromagnetic transition temperature *T*_C_ and the thermal hysteresis Δ*T*_hys_ can be obtained. The values of *T*_C_ and Δ*T*_hys_ were determined from the maximum in the first derivative of the heating and cooling curves in an applied magnetic field of 0.01 T

## 3. Results

### 3.1. Structure

**Figure 1:**
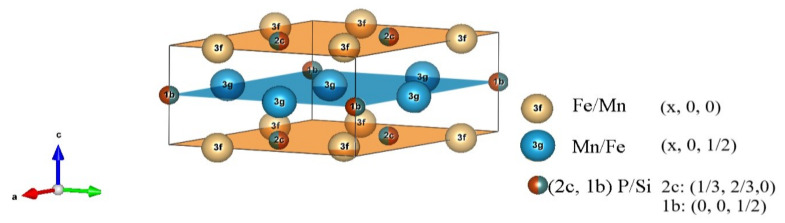
Unit cell of the hexagonal Fe_2_P structure (space group *P-62m*) indicated is the layered structure with the Wyckoff positions. The Mn and Fe atoms occupy the *3g* and *3f* positions (Mn prefers the *3g* site and Fe the *3f* site), while the P and Si atoms occupy the *2c* and *1b* positions.

A structural analysis has been performed to refine the lattice structure of the Mn*_x_*Fe_2−*x*_P_1−*y*_Si*_y_* quaternary compounds. Four possible lattice structures are found in the Mn*_x_*Fe_2−*x*_P_1−*y*_Si*_y_* compounds. The orthorhombic Co_2_P structure (*Pnma*) forms in the Si poor region (*y* < 0.15). The single hexagonal Fe_2_P structure (*P-62m*) of Figure 1 is found for Si compositions of 0.24 ≤ *y* ≤ 0.5. For a Si composition of 0.5 ≤ y < 1.0, a three-phase region is observed, which consists of the Fe_2_P structure, the hexagonal Mn_5_Si_3_ structure (*P63/mcm*) and the cubic Fe_3_Si structure (*Fm-3m*) [34].

A silicon concentration range of 0.33 ≤ *y* ≤ 0.6 was chosen to synthesise samples Mn*_x_*Fe_2−*x*_P_1−*y*_Si*_y_* with a hexagonal Fe_2_P lattice structure. The investigated range of silicon concentrations has been restricted to avoid the appearance of the orthorhombic lattice structure when the silicon concentration is too low. In contrast, if the silicon concentration is too high, the three-phase region is entered (with the Fe_2_P-type main phase and Fe_3_Si-type and Mn_5_Si_3_-type impurity phases), resulting in a decrease in the phase fraction of the main phase [34].

**Figure 2:**
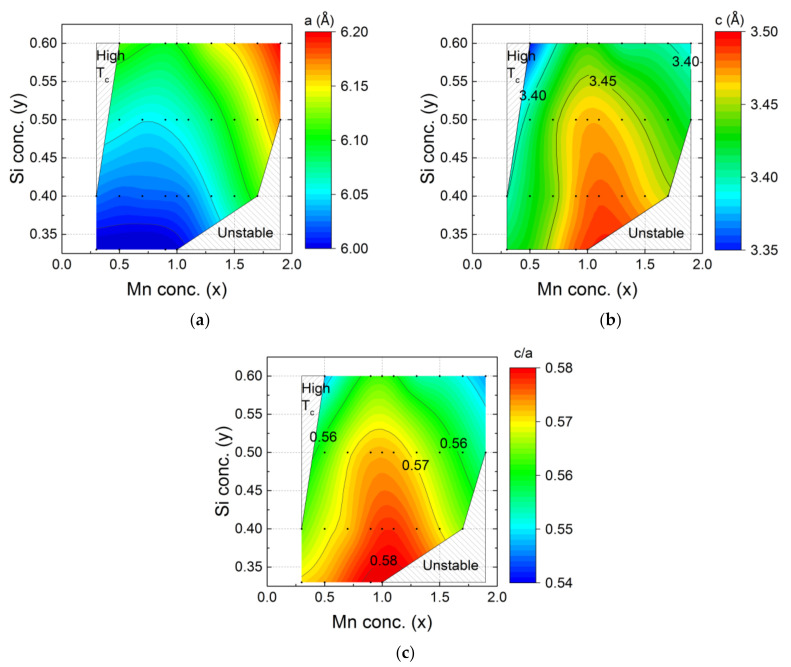
Composition dependence of the lattice parameters for the hexagonal Fe_2_P-type main phase in the Mn*_x_*Fe_2−*x*_P_1−*y*_Si*_y_* system with (**a**) lattice parameter *a*, (**b**) lattice parameter *c* and (**c**) the *c/a* ratio. The black points correspond to the experimental data. The colour code is obtained from linear interpolation. As the *c/a* ratio may change discontinuously at the first order ferromagnetic phase transition, all data were measured in the paramagnetic state. In Table 1.

In Figure 2 the evolution of the lattice parameters *a* and *c* in the hexagonal main phase of the Mn*_x_*Fe_2−*x*_P_1−*y*_Si*_y_* compounds is shown as a function of composition, refined values are given in Table 1. When *x* > 1 the lattice parameter *a* expands for increasing Mn and Si concentrations (Figure 2a). This phenomenon can be explained in terms of the atomic radius. The manganese atom has a larger radius than the Fe atom, and Mn prefers the crystallographic *3g* site, while Fe prefers the *3f* site. When the *3g* site is fully occupied, then the rest of the Mn atoms occupy the *3f* site [30]. As a result, the lattice parameter *a* expands.

Figure 2b indicates that the lattice parameter *c* has a maximum value when the metal ratio is Mn:Fe = 1:1 (corresponding to x = 1) In this case, the *3g* site is fully occupied by Mn and the *3f* site is fully occupied by Fe. The lattice parameter *c* is found to shrink for increasing Fe, Mn or Si concentration. The *c/a* ratio ranges from 0.54 to 0.58 and shows a similar dependence on composition as lattice parameter *c* (Figure 2c).

### 3.2. Magnetic Properties of Mn_x_Fe_2−__x_P_1−__y_Si_y_

**Figure 3:**
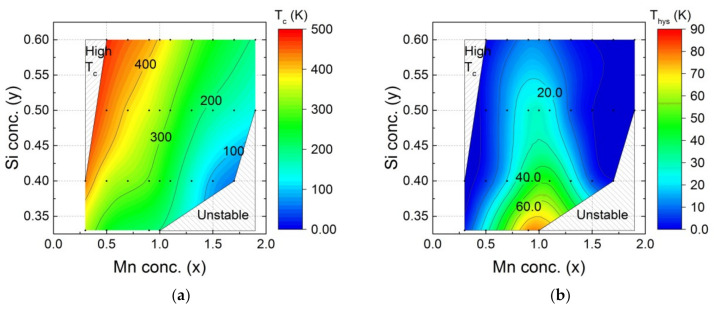
Magnetic properties of Mn*_x_*Fe_2−*x*_P_1−*y*_Si*_y_* (*x* = 0–2, *y* = 0.33–0.60) compounds showing the composition dependence of (**a**) the ferromagnetic transition temperature *T*_C_ and (**b**) the thermal hysteresis Δ*T*_hys_. The black points correspond to the experimental data. The colour code is obtained from linear interpolation.

The ferromagnetic-to-paramagnetic transition temperature *T*_C_ and the thermal hysteresis in this transition Δ*T_hys_* for the hexagonal Fe_2_P-type main phase in the Mn*_x_*Fe_2−*x*_P_1−*y*_Si*_y_* (*x* = 0.3–2.0, *y* = 0.33–0.60) compounds was investigated by magnetisation measurements and is shown in Figure 3. The ferromagnetic transition temperature *T*_C_ increases with increasing Fe and Si contents. Materials with a low thermal hysteresis Δ*T*_hys_ can be found for both Fe-rich and Mn-rich compositions.

#### 3.2.1. Transition Temperature and Thermal Hysteresis

The Mn*_x_*Fe_2−*x*_P_1−*y*_Si*_y_* compounds cover a broad range of ferromagnetic transition temperatures [36]. In this work, the experimental transition temperature *T*_C_ was found to range from 65 to 470 K. The highest ferromagnetic transition temperature appears in the compound Mn_0.3_Fe_1.7_P_0.6_Si_0.4_ (*T*_C_ = 470 K). This transition temperature is too high for magnetic refrigeration, magnetic heat pumping or waste heat conversion applications near room temperature. According to the trend of the phase diagram, it is clear that the transition temperature can be tuned higher if we increase the Si concentration. Therefore, part of the phase diagrams is marked ‘High *T*_C_’ as it shows a ferromagnetic transition temperature beyond the application range (*T*_C_ > 470 K). The compounds Mn_1.7_Fe_0.3_P_0.6_Si_0.4_ shows the lowest ferromagnetic transition temperature (*T*_C_ = 65 K) in the Mn*_x_*Fe_2−*x*_P_1−*y*_Si*_y_* phase diagram. In general, the highest transition temperatures are found for low Mn and high Si contents, while the lowest are found for low Fe and Si contents.

The thermal hysteresis Δ*T*_hys_ covers a broad range of values from 0 to 90 K. It is interesting to note that the largest hysteresis was found for a metal ratio of Fe:Mn ≈ 1:1 (*x* ≈ 1), corresponding to a full occupancy of the *3g* site by Mn and the *3f* site by Fe. The thermal hysteresis decreases with decreasing Fe, Mn or P content. The thermal hysteresis of the Mn*_x_*Fe_2−*x*_P_1−*y*_Si*_y_* compounds is further tuneable by optimising the stoichiometry [37].

#### 3.2.2. Unstable Compositions

**Figure 4:**
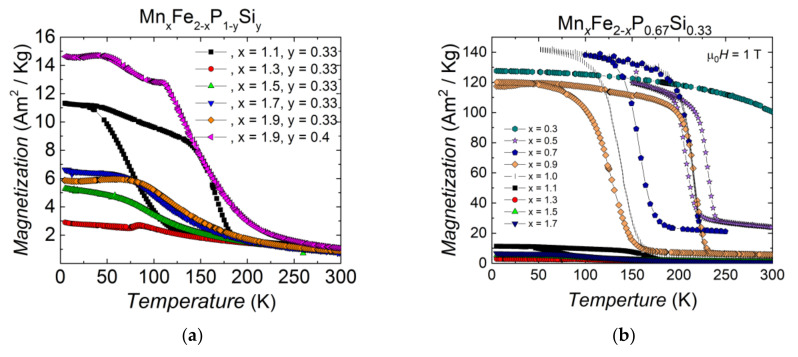
Magnetisation in 1 T as a function of temperature for (**a**) Mn*_x_*Fe_2−*x*_P_1−*y*_Si*_y_* compounds in the ‘unstable’ zone and (**b**) Comparison of ferromagnetic and ‘unstable’ samples in Mn*_x_*Fe_2−*x*_P_0.67_Si_0.33_ compounds.

In Figure 3, there is an area in the compositional maps marked as ‘Unstable’. Figure 4a shows examples of the magnetisation as a function of temperature for Mn*_x_*Fe_2−*x*_P_1−*y*_Si*_y_* compounds in the unstable region (*x* = 1.1–1.9, *y* = 0.33 and *x* = 1.6, *y* = 0.4). Compared to ferromagnetic Fe_2_P-type samples (shown in Figure 4b), these samples generally show more than one magnetic transition, or they undergo an antiferromagnetic transition from the paramagnetic state instead of a ferromagnetic transition, while normal Fe_2_P-type samples have one magnetic phase transition with a magnetisation value above 120 Am^2^/kg under 1 T external magnetic field. These transitions cannot be used in magnetic refrigerators nor for thermomagnetic motors. Therefore, they are not studied further.

## 4. Discussion

### 4.1. Impurity Phase

**Figure 5:**
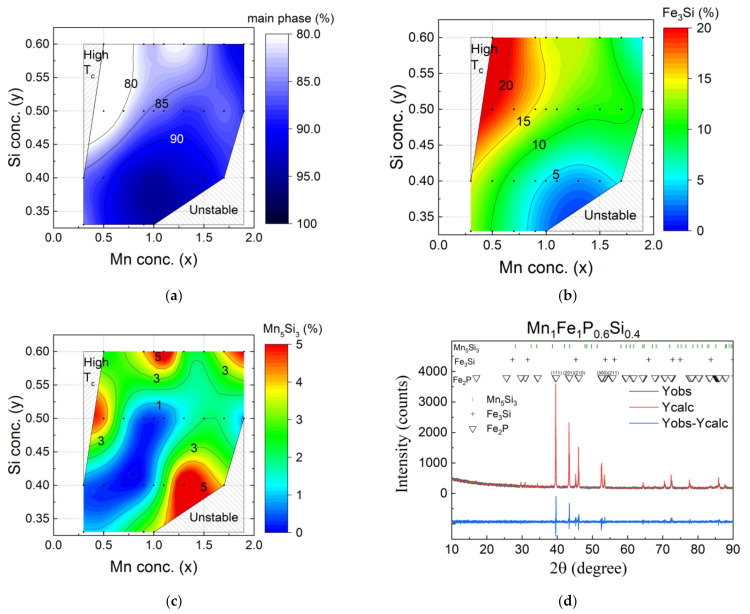
Phase stability diagrams of the Mn*_x_*Fe_2−*x*_P_1−*y*_Si*_y_* (*x* = 0.3–2.0 and *y* = 0.33–0.60) system showing the phase fractions of (**a**) the Fe_2_P-type main phase, (**b**) the Fe_3_P-type impurity phase and (**c**) the Mn_5_Si_3_-type impurity phase. The black points correspond to the experimental data. The colour code is obtained from linear interpolation. (**d**) XRD pattern of MnFeP_0.6_Si_0.4_ with measured and calculated intensities.

The silicon concentration (0.33 ≤ *y* ≤ 0.6) was chosen to synthesise Mn*_x_*Fe_2−*x*_P_1−*y*_Si*_y_* samples with a hexagonal Fe_2_P-type lattice structure. The investigated range of silicon concentrations has been restricted to avoid the appearance of the orthorhombic lattice structure when the silicon concentration is too low. In contrast, if the silicon concentration is too high, a three-phase region is entered (consisting of the Fe_2_P-type main phase and the Fe_3_Si-type and Mn_5_Si_3_-type impurity phases), resulting in a decrease in the phase fraction of the main phase [34]. The phase stability diagrams for the phase composition of the Fe_2_P-type main phase and the Fe_3_Si-type [27] and Mn_5_Si_3_-type impurity phases are shown in Figure 5. The main impurity phase in the iron-rich Mn*_x_*Fe_2−*x*_P_1−*y*_Si*_y_* compounds (*x* < 1) is the Fe_3_Si-type phase. Figure 5b indicates that the Fe_3_Si-type impurity increases continuously for increasing Fe and Si concentrations. For high Mn and low Si concentrations, the Mn_5_Si_3_-type impurity phase is dominant (Figure 5c).

**Figure 6:**
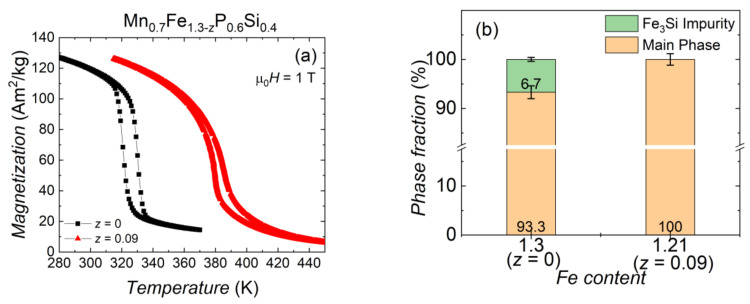
(**a**) Magnetisation as a function of temperature for Mn_0.7_Fe_1.3−*z*_P_0.6_Si_0.4_ for *z* = 0.00 and 0.09 sample, measured in an applied magnetic field of 1 T. (**b**) The phase fraction of the Mn_0.7_Fe_1.3−*z*_P_0.6_Si_0.4_ for *z* = 0.00 and 0.09 sample obtained from X-ray diffraction.

By optimising the stoichiometry, the transition temperature *T*_C_ and the thermal hysteresis Δ*T*_hys_ can change significantly. As shown in Figure 6, in Mn_0.7_Fe_1.3−*z*_P_0.6_Si_0.4_ a change in the Fe content from 1.30 (*z* = 0.00) to 1.21 (*z* = 0.09) results in an increase in transition temperature of 52 K and a decrease in thermal hysteresis of 10 K. These changes are caused by a variation in the amount of impurity phase and a shift in Mn, Fe ratio and P, Si ratio [12]. From the X-ray refinement, the nominally stoichiometric sample Mn_0.7_Fe_1.3_P_0.6_Si_0.4_ has about 6% Fe_3_Si impurity. (Figure 6b) The impurity phase can be removed by optimising the stoichiometry, which is accompanied by an increase in *T*_C_. Considering this behaviour, it is better to start the material synthesis with a *T*_C_ below the desired working temperature and then adjust it by reducing impurities.

### 4.2. Heat of Transformation and Thermal Hysteresis as a Function of Structural Parameters

The heat of transformation of the magnetic phase transition and thermal hysteresis are two intuitive indicators that can reveal the usability of a material for magnetocaloric applications. Often, a first order magnetic phase transition (FOMT) is associated with a large heat of transformation and a large hysteresis. For applications we like to have large magnetic entropy changes that can be boosted by contributions from heat of transformation, at the same time thermal hysteresis exceeding the adiabatic temperature change can’t be utilized in simple magnetisation and demagnetisation cycles. Therefore, these two parameters as important characteristics are compared to *c/a* ratio in this work.

**Figure 7:**
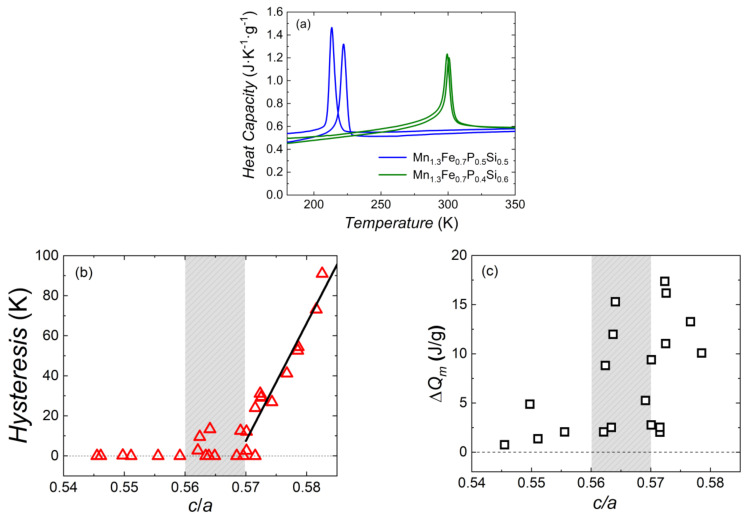
(**a**) Temperature dependence of the specific heat of sample Mn_1.3_Fe_0.7_P_0.5_Si_0.5_ and Mn_1.3_Fe_0.7_P_0.4_Si_0.6_ measured in zero fields upon cooling and heating. Thermal hysteresis (**b**) and heat of magnetic transformation Δ*Q_m_* (**c**) as a function of the *c/a* ratio for the lattice parameters of the hexagonal Fe_2_P-type main phase of the Mn*_x_*Fe_2−*x*_P_1−*y*_Si*_y_* system.

Figure 7a shows the temperature dependence of the specific heat and large entropy change at the magnetic phase transition of samples Mn_1.3_Fe_0.7_P_0.5_Si_0.5_ and Mn_1.3_Fe_0.7_P_0.4_Si_0.6_. These two samples both show a relatively large sharp peak, and the heat of magnetic transformation Δ*Q_m_* can be obtained by integrating the peak area. Note that this specific-heat peak contains all thermal effects involved in the magnetic phase transition. The Δ*Q_m_* of Mn_1.3_Fe_0.7_P_0.5_Si_0.5_ and Mn_1.3_Fe_0.7_P_0.4_Si_0.6_ compounds is 4.72 and 4.58 (J g^−1^), respectively.

A clear correlation becomes visible when the thermal hysteresis is compared to the *c/a* ratio of the lattice parameters for the Fe_2_P-type hexagonal main phase of the Mn*_x_*Fe_2−*x*_P_1−*y*_Si*_y_* system observed. As shown in Figure 7b, the lowest *c/a* ratio starts from 0.5455 (Mn_0.5_Fe_1.5_P_0.4_Si_0.6_) and ends at 0.5826 (Mn_0.9_Fe_1.1_P_0.67_Si_0.33_). Most samples display no hysteresis when the *c/a* ratio is smaller than 0.57. For instance, sample Mn_1.5_Fe_0.5_P_0.6_Si_0.4_ is a sample without thermal hysteresis (*c/a =* 0.5696) and sample Mn_0.5_Fe_1.5_P_0.6_Si_0.4_ is a sample with a 2.6 K thermal hysteresis (*c/a =* 0.5701). In the grey region 0.56 < *c/a* < 0.57 we find both samples with and without hysteresis, and when the *c/a* ratio is larger than 0.57a steep increase in hysteresis with increasing *c/a* ratio. For instance, sample Mn_0.7_Fe_1.3_P_0.5_Si_0.5_ is a FOMT sample with about 12 K thermal hysteresis (*c/a =* 0.5701). The red line is a linear fit to these data.

In Figure 7c the heat of magnetic transformation is compared to the *c/a* ratio of the lattice parameters (Figure 2c) for the Fe_2_P-type hexagonal main phase for the Mn*_x_*Fe_2−*x*_P_1−*y*_Si*_y_* system. There is no clear trend that these two parameters are related.

In general, thermal hysteresis is controlled by the phase nucleation, which is a kinetic process, and the *c/a* ratio is a parameter of the crystal structure. The reason why these two parameters correlated is not directly obvious. It could be that the hysteresis is related to the unit-cell distortion when the transition is crossed. This interesting experimental relationship between thermal hysteresis and the *c/a* ratio deserves further study.

### 4.3. Suitable Materials for Magnetic Energy Conversion Applications

**Figure 8:**
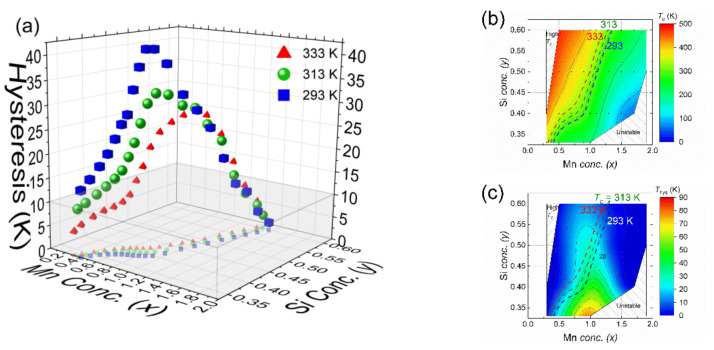
(**a**) Concentration dependence of the thermal hysteresis in the Mn*_x_*Fe_2−*x*_P_1−*y*_Si*_y_* system for transition temperatures *T_C_* of 293, 313 and 333 K. The points correspond to interpolated values in the magnetic diagram. The blue and red symbols and lines mark the lower and upper temperature limit of the optimal cycling range between 20 °C (293 K) and 60 °C (333 K). The green symbols and line mark the considered optimal *T*_C_ of 40 °C (313 K). Schematic graph with contour lines in phase diagram of Mn*_x_*Fe_2−*x*_P_1−*y*_Si*_y_* (*x* = 0–2, *y* = 0.33–0.60) compounds showing the composition dependence of (**b**) the ferromagnetic transition temperature *T*_C_ and (**c**) the thermal hysteresis Δ*T*_hys_.

The structural, magnetic and phase stability diagrams allow us to identify proper candidates for each application in different working temperature range. Based on this working temperature window, contour lines can be drawn in the magnetic diagram for *T*_C_ (Figure 8b) and duplicate them at the same positions in the diagram for the thermal hysteresis (Figure 8c). The obtained information on the transition temperature and thermal hysteresis is summarised in Figure 8a. In Figure 8a, the *x*, *y* axis represents the Mn and Si concentration, respectively, The z axis represents thermal hysteresis obtained from the thermal contour line drawn in the thermal hysteresis part of the phase diagram (Figure 8c). These 3D concentration dependence of the thermal hysteresis graph has projections on the xy plane, which are the same as the contour lines shown in the phase diagram.

As an example for the working temperature range of a thermomagnetic motor beeing between 20 °C (293 K) and 60 °C (333 K), with an optimal *T*_C_ of 40 °C (313 K). For each temperature, the hysteresis increases with increasing Mn and Si concentration. Then, the hysteresis drops after it reaches a peak for a Mn content of *x* ≈ 1.0 and a Si content of *y* ≈ 0.45. Promising candidates can only be found in the grey area in Figure 8a, which correspond to the Fe-rich/P-rich part (*x* < 0.7, *y* < 0.4) and the Mn-rich/Si-rich part (*x* > 1.1, *y* > 0.5) of the phase diagram.

## 5. Conclusions

A full-range magnetic phase diagram of the Mn*_x_*Fe_2−*x*_P_1−*y*_Si*_y_* system has been established as a guide to find suitable materials for energy conversion applications in a thermomagnetic motor. We find a strong correlation between thermal hysteresis and *c/a* ratio, however, there are a wide range of samples with low (<2 K) or absent hysteresis that yet display large heat of magnetic transformation. Both Mn-rich with a high Si and Fe-rich samples with a low Si concentration were found to show a low hysteresis that can form promising candidates for applications in a thermomagnetic motor. It appears that Mn-rich samples are most suited for applications well below room temperature, while Mn-poor samples can be utilised at higher temperatures. The interesting experimental relationship between thermal hysteresis and the *c/a* ratio deserves further study. Furthermore, reducing the impurity phase fraction by optimising stoichiometry is important in lowering thermal hysteresis.

## Figures and Tables

**Table 1:** Properties of compounds in the phase diagram of Mn*_x_*Fe_2−*x*_P_1−*y*_Si*_y_* system.

Mn Conc.	Si Conc.	T_C_	T_hys_	Heat of Trans-Formation	a	c	c/a	Main Phase %		Fe_3_Si Phase %		Fe_5_Si_3_ Phase	
(*x*)	(*y*)	(K)	(K)	(J/g)	(Å)	(Å)						%	
0.3	0.33	362.3	0.0	2.0	5.99145(3)	3.42440(3)	0.5715	84.0	±0.8	14.8	±0.3	1.3	±0.4
0.5	0.33	224.3	23.9	- ^1^	5.99147(5)	3.42442(3)	0.5715	90.4	±1.4	8.7	±0.3	1.0	±0.1
0.7	0.33	189.6	76.6	- ^1^	5.97865(6)	3.47447(4)	0.5811	89.7	±1.3	9.0	±0.4	1.3	±0.2
0.9	0.33	214.5	91.0	- ^1^	5.98880(4)	3.48886(3)	0.5826	92.1	±1.5	3.9	±0.3	4.0	±0.7
1.0 *	0.33	211.7	73.1	- ^1^	5.99761(4)	3.48817(3)	0.5816	95.8	±1.1	1.4	±0.2	2.8	±0.2
0.3	0.4	469.4	2.7	2.5	6.02914(5)	3.39681(4)	0.5634	88.2	±1.2	11.7	±0.4	0.1	±0.1
0.5	0.4	324.0	2.6	2.8	6.01422(5)	3.42870(4)	0.5701	86.5	±1.1	13.2	±0.4	0.3	±0.1
0.7	0.4	327.0	12.5	5.2	6.02434(5)	3.45378(3)	0.5733	93.7	±1.1	6.3	±0.4	0.3	±0.1
0.9	0.4	307.1	41.2	13.3	6.02771(4)	3.47656(8)	0.5768	93.3	±1.3	6.7	±0.4	-	-
1.0	0.4	282.2	56.4	10.1	6.01853(7)	3.48194(5)	0.5785	96.0	±1.6	3.4	±0.3	0.6	±0.1
1.1	0.4	219.6	54.4	- ^1^	6.02441(5)	3.48591(4)	0.5786	94.4	±1.1	4.3	±0.2	1.3	±0.2
1.3	0.4	146.9	26.9	- ^1^	6.05270(5)	3.47604(4)	0.5743	90.3	±1.2	0.2	±0.0	9.5	±0.6
1.5	0.4	73.9	0.0	- ^1^	6.08221(4)	3.46432(3)	0.5696	94.7	±1.2	1.6	±0.4	3.7	±0.1
1.7 *	0.4	64.9	0.0	- ^1^	6.11159(5)	3.45225(3)	0.5649	86.9	±2.4	9.2	±2.6	3.9	±0.2
0.5	0.5	443.3	2.7	2.0	6.06083(6)	3.40681(4)	0.5621	72.2	±2.6	21.5	±0.8	6.3	±3.3
0.7	0.5	373.0	10.9	9.4	6.04712(8)	3.44757(6)	0.5701	81.3	±1.7	18.1	±0.7	0.6	±0.2
0.9	0.5	362.7	29.5	16.2	6.05370(6)	3.46627(5)	0.5726	84.2	±1.4	15.7	±0.5	0.2	±0.1
1.0	0.5	348.6	31.1	17.4	6.06062(4)	3.46886(3)	0.5724	87.4	±0.9	12.2	±3.4	0.4	±0.1
1.3	0.5	219.1	0.0	- ^1^	6.08292(5)	3.45815(4)	0.5685	89.5	±1.5	9.2	±0.2	1.3	±0.2
1.5	0.5	182.7	0.3	- ^1^	6.11228(3)	3.44680(3)	0.5639	88.7	±1.0	9.2	±0.3	2.1	±0.1
1.7	0.5	162.5	0.0	- ^1^	6.14091(5)	3.43373(4)	0.5592	83.2	±1.1	14.4	±0.6	2.4	±0.2
1.9 *	0.5	142.6	2.0	- ^1^	6.17221(5)	3.41687(3)	0.5536	89.3	±1.0	10.7	±0.7	-	-
0.5	0.6	473.9	0.0	0.7	6.12222(5)	3.33965(4)	0.5455	71.9	±0.8	26.3	±0.5	1.8	±0.2
0.7	0.6	443.6	4.4	15.4	6.13927(5)	3.34006(4)	0.5440	79.6	±1.0	17.8	±0.4	2.6	±0.2
0.9	0.6	421.9	15.4	12.0	6.09298(8)	3.43456(6)	0.5637	81.0	±1.5	14.6	±0.6	4.4	±0.4
1.0	0.6	403.0	13.4	15.3	6.09631(5)	3.43886(4)	0.5641	90.6	±1.2	8.1	±0.4	1.3	±0.1
1.1	0.6	364.3	15.2	8.8	6.1067(1)	3.4357(9)	0.5626	81.5	±1.3	16.2	±0.5	2.3	±0.2
1.3	0.6	302.1	0.2	4.9	6.1563(1)	3.38444(9)	0.5497	79.7	±1.3	20.1	±0.5	0.3	±0.1
1.5	0.6	255.2	0.0	2.0	6.14830(5)	3.41576(3)	0.5556	83.1	±1.1	15.0	±0.5	1.9	±0.1
1.7	0.6	225.8	1.3	1.4	6.17386(8)	3.40240(5)	0.5511	90.8	±1.1	4.9	±0.0	4.4	±0.2
1.9	0.6	191.6	0.4	- ^1^	6.20058(7)	3.38632(4)	0.5461	92.1	±1.8	2.2	±0.3	5.7	±0.6

^1^ outside the measurement range of DSC, * at edge to unstable.

## Data Availability

The data are available upon reasonable request.
